# Sorption of Interleukins 12 and 23 Using the Developed Magnetocontrollable Ustekinumab-Based Sorbent

**DOI:** 10.17691/stm2020.12.4.09

**Published:** 2020-08-27

**Authors:** I.P. Gontar, O.I. Emelyanova, O.A. Rusanova, I.A. Zborovskaya

**Affiliations:** Professor, Head of the Laboratory of Clinical Immunology; Research Institute of Clinical and Experimental Rheumatology named after A.B. Zborovsky, 76 Zemlyachki St., Volgograd, 400138, Russia; Leading Researcher; Research Institute of Clinical and Experimental Rheumatology named after A.B. Zborovsky, 76 Zemlyachki St., Volgograd, 400138, Russia; Junior Researcher; Research Institute of Clinical and Experimental Rheumatology named after A.B. Zborovsky, 76 Zemlyachki St., Volgograd, 400138, Russia; Professor, Director Research Institute of Clinical and Experimental Rheumatology named after A.B. Zborovsky, 76 Zemlyachki St., Volgograd, 400138, Russia

**Keywords:** interleukin-12, IL-12, interleukin-23, IL-23, psoriatic arthritis, antibodies, magnetosorbents, sorption.

## Abstract

**The aim of the study** was to assess the sorption capacity of Ustekinumab, a drug based on interleukins 12 (IL-12) and 23 (IL-23), when using it with a magnetocontrollable sorbent.

**Materials and Methods.:**

To reduce the concentration of proinflammatory cytokines (IL-12, IL-23), the blood of patients with psoriatic arthritis was passed through magnetocontrollable polyacrylamide granules (MPG) of spherical shape with the particle size of 10–100 μm, obtained by emulsion polymerization. Perfusion was performed through a 10 ml column equipped with an electromagnet, into which MPG with immobilized antibodies to IL-12 and IL-23 was added. Ustekinumab, a commercial drug with concentration of monoclonal antibodies equaling 0.2 mg per 1 ml of saline was used as the source of antibodies to these interleukins. The specific sorption capacity of MPG was determined using a column filled with the granules in a volume of 0.2 ml and inducing their subsequent interaction with 1 ml of IL-12 and IL-23 solutions with increasing concentrations.

The heparinized blood of 10 patients with psoriatic arthritis of various degrees of activity, who had no parenteral administration of IL-12/IL-23 inhibitors (Ustekinumab) for 12 months, was subjected to *in vitro* treatment. The blood of 10 healthy donors was used as the control; the perfusion procedure was similar.

**Results.:**

Digital parameters were measured for each sample before and after the interaction between the sorbent and blood plasma. There was a significant decrease in cytokines from the baseline — by 99.8% (in donors) and by 99.9% (in patients). When using a carbon sorbent, their concentration decreases by 92.6% from the baseline. As a result of sorption, blood cell counts did not change reliably, which is an additional positive aspect of this procedure.

**Conclusion.:**

The maximum possible decrease in the level of cytokines has great practical importance, since they play a leading role in the pathogenesis of psoriatic arthritis. The use of a synthesized Ustekinumab-based sorbent is a highly effective way of removing them simultaneously from blood plasma.

## Introduction

Protein regulators as growth mediators involved in the differentiation of lymphoid and mesenchymal cells and immune inflammation cells are known to play the leading role in the pathogenesis of many rheumatic autoimmune diseases, including psoriatic arthritis. The immune system cells, such as fibroblasts, platelets, monocytes, macrophages synthesize cytokines, interleukins, interferons, and other factors of immune inflammation [1–3].

In psoriatic arthritis and other immune diseases, there is pathological synthesis of interleukins 12 (IL-12) and 23 (IL-23). The increased IL-12 content is synchronized with the amount of IL-1, IL-2, IL-10, interferon-α (IFN-α), and tumor necrosis factor-α (TNF-α). Their increased level is found in the synovial tissue.

These interleukins are heterodimeric cytokines containing two subunits of glycosylated proteins connected by disulfide bonds and having the common p40 subunit. When it interacts with the p35 subunit, IL-12 is formed, while interaction with the p19 subunit results in formation of IL-23. These cytokines are synthesized mainly by macrophages, dendritic cells. They produce their biological action by fixing on double-stranded heterodimeric receptor complexes located on the surface of CD4^+^ T lymphocytes and natural killer cells (NK cells). IL-23 stimulates Th17-lymphocytes that begin to produce proinflammatory factors, including IL-17, and these induce synthesis of other proinflammatory agents, according to the feedback principle [4–7].

In recent years, fundamentally novel anti-inflammatory drugs have been created using biotechnology methods. They are genetically engineered biological agents, the use of which has made it possible to increase the efficacy of pharmacotherapy for rheumatic diseases significantly. These agents include monoclonal antibodies to certain determinants of immunocompetent cells, or proinflammatory cytokines, and hybrid protein molecules inhibiting cytokine activity or interaction between T and B lymphocytes. The main advantage of these drugs is their high specificity providing a selective effect on certain links in the pathogenesis of immune-inflammatory diseases and affecting the normal mechanisms of the immune system at a minimum [8].

Today, parenteral administration of genetically engineered biological drug Ustekinumab (Cilag AG, Switzerland), IL-12 and IL-23 inhibitor, based on monoclonal antibodies to IL-12 and IL-23, is widely used to treat psoriatic arthritis [8, 9]. This commercial drug is an interleukin inhibitor representing human IgG1k monoclonal antibodies produced by a recombinant cell line and subjected to multi-stage purification, including inactivation and removal of viral particles. High biological affinity and specificity of the drug for the p40 subunit of human interleukins IL-12 and IL-23 provides a blocking effect and prevents their active interaction with the IL-12Rβ1 receptor and the p40 subunit, while simultaneously neutralizing both molecules. This drug quickly reduces disease activity, inhibits destruction of the affected joints. However, side effects (frequent allergic and infusion reactions) and the high cost limit its use for the same patient. Elimination of the studied interleukins from the bloodstream with magnetocontrollable sorbents is a convenient pathogenetic tool for suppressing their activity in autoimmune diseases. Cytokines can be removed using a carbon sorbent with a surface locally modified with aminocaproic acid [10]. In this case, cytokine sorption from blood serum is carried out through a solid-phase carrier, the carbon adsorbent surface modified with aminocaproic acid with its subsequent polycondensation. To reduce the concentration of proinflammatory cytokines IL-12 and IL-23, the authors propose passing the blood of patients with psoriatic arthritis through granules obtained previously by means of emulsion polymerization [11]. The commercial drug Ustekinumab was the source of antibodies to interleukins [12].

**The aim of the study** was to assess the sorption capacity of Ustekinumab, a drug based on interleukins 12 and 23, when using it with a magnetocontrollable sorbent.

## Materials and Methods

The heparinized blood of 10 patients with psoriatic arthritis of various degrees of activity, who had no parenteral administration of cytokine inhibitors for 12 months, was subjected to *in vitro* treatment. The blood of 10 healthy donors was used as the control; the perfusion procedure was similar.

Ustekinumab with the concentration of monoclonal antibodies of 0.2 mg per 1 ml of saline was used as the source of antibodies to interleukins IL-12 and IL-23 immobilized on granules of spherical shape with a particle size of 10–100 μm. After completing the polymerization, the granules were washed.

The specific sorption capacity of magnetocontrollable polyacrylamide granules (MPG) was determined as follows. A mixture of IL-12 and IL-23 solutions (1 ml) with increasing concentrations was passed through a mini-column with a 0.2 ml working chamber filled with MPG. After perfusion of each variant of the solutions, MPG was replaced, the loop was washed with 1 M Glycine-HCI buffer, pH 2.2, and standard phosphate-buffered saline.

The concentration of IL-12 and IL-23 in desorbed solutions was determined by ELISA using commercial kits (Bender MedSystems, USA, for IL-12 and Bender MedSystems, Austria, for IL-23). When studying the resulting solutions of IL-12 and IL-23 with initial concentrations of 5, 10, 50, 100, 500, 1500 pg/ml after their perfusion through MPG, interleukins were actually undetectable.

**Statistical data processing** was performed using Statistica 6.0, Excel 5.0, Statgraphics 3.0, SPSS 12.0 software packages. Sample characteristics were presented as mean (M) and standard deviation (σ). Student’s t-test was used to assess the quantitative differences in different groups. The results obtained were evaluated using the p value as the estimated probability that the effect obtained in the study was random. Sample differences were considered statistically significant at p<0.05.

## Results and Discussion

The dynamics of plasma interleukin concentrations in donors and patients with psoriatic arthritis is shown in Table 1. It demonstrates that during perfusion through a magnetocontrollable sorbent, a statistically significant decrease in the content of IL-12 and IL-23 as compared to the baseline value was achieved in both groups, patients and healthy individuals. In this case, the maximum possible decrease in the level of cytokines is of great practical importance, since they play a leading role in the pathogenesis of psoriatic arthritis. When using the carbon sorbent described in the literature [10], their concentration decreases by 92.6% compared to the initial level.

**Table 1:** Change in IL-12 and IL-23 concentrations resulting from perfusion through magnetocontrollable polyacrylamide granules with immobilized antibodies to IL-12 and IL-23 (%) (M±σ)

Group	IL-12		IL-23	
	Baseline	After perfusion	Baseline	After perfusion
Healthy donors (n=10)	63.23±14.17	0.04±0.02	50.12±12.16	0.03±0.01
	(—)	(99.8)	(—)	(99.9)
Patients with psoriatic arthritis (n=10)	85.16±39.28	0.02±0.01	70.53±28.14	0.04±0.01
	(—)	(99.9)	(—)	(99.9)

Note: statistical significance of differences in values before and after perfusion, p<0.001 in all cases (the percentage increase in concentration is indicated in parentheses).

Blood perfusion was performed through the sorbent in a 10 ml column equipped with an electromagnet, into which MPG with immobilized antibodies to IL-12 and IL-23 was added. 20 ml of native heparinized blood was perfused through the column at a rate of 10 ml/h. On completing the perfusion, unbound proteins were removed from MPG by washing with a buffered saline solution (pH 7.4) and regeneration was performed with 1 M glycine-HCI buffer pH 2.2 for 10 min. After the first regeneration, loss in sorption capacity was 15%. During subsequent repeated regenerations (9 times), no statistically significant changes occurred in the specific sorption capacity.

Blood plasma concentration of cytokines IL-12 and IL-23 was determined by ELISA using commercial Bender MedSystems kits, blood cell counts (erythrocytes, leukocytes, platelets) were also assessed. The values of all studied parameters were measured for each sample twice, before and after perfusion.

When cytokines were removed, blood cell counts did not change significantly, which is an additional advantage of the proposed perfusion method (Table 2).

**Table 2:** Changes in blood cell counts resulting from perfusion through magnetocontrollable polyacrylamide granules with immobilized antibodies to IL-12 and IL-23 (M±σ)

Parameter	Baseline (n=10)	After perfusion (n=10)
Erythrocytes (×10^12^/L)	3.80±0.07	3.70±0.06
Leukocytes (×10^9^/L)	5.90±0.21	5.60±0.21
Platelets (×10^9^/L)	250.0±8.89	245.0±8.41

Note: statistical significance of differences in values before and after perfusion as compared to the baseline data, р≤0.05.

## Conclusion

The results of IL-12 and IL-23 sorption using the magnetocontrollable sorbent synthesized by us on the basis of polyacrylamide gel and Ustekinumab indicate highly effective removal of pathogenetic cytokines. This sorbent has low damage rate in relation to blood cells and low non-specific sorption. Extracorporeal removal of cytokines from the bloodstream is likely to be a promising preparatory stage in treatment of patients with rheumatic diseases with genetically modified drugs. Similar procedures with the use of commercial antibody agents based on specific monoclonal immunoglobulins can be used in a possible “cytokine storm” in the pathogenesis of autoimmune diseases and in the therapy of other rheumatic diseases.
